# What Helps and What Hinders the Creation of a Smoke-free Home: A Qualitative Study of Fathers in Scotland

**DOI:** 10.1093/ntr/ntab228

**Published:** 2021-11-10

**Authors:** Rachel O’Donnell, Peter McCulloch, Lorraine Greaves, Sean Semple, Amanda Amos

**Affiliations:** 1 Institute for Social Marketing and Health, Faculty of Health Sciences and Sport, University of Stirling, Stirling, FK9 4LA, UK; 2 School of Health Sciences, University of Dundee, Dundee, DD1 4HN, UK; 3 Centre of Excellence for Women’s Health & School of Population and Public Health, University of British Columbia, Vancouver, Canada; 4 Usher Institute, University of Edinburgh, Edinburgh, EH8 9AG, UK

## Abstract

**Introduction:**

Few studies have explored fathers’ views and experiences of creating a smoke-free home, with interventions largely targeting mothers. This study aimed to identify barriers and facilitators to fathers creating a smoke-free home, to inform future intervention development.

**Methods:**

Eighteen fathers who were smokers and lived in Scotland were recruited from Dads’ community groups, Early Years Centres and through social media advertising. Semi-structured interviews explored their views and experiences of creating a smoke-free home. A theory-informed thematic analysis using the COM-B model highlighted ways in which capability, opportunity, and motivations shaped fathers’ home smoking behaviors.

**Results:**

Several fathers understood the health risks of second-hand smoke exposure through public health messaging associated with recent smoke-free legislation prohibiting smoking in cars carrying children. Limited understanding of effective exposure reduction strategies and personal mental health challenges reduced some fathers’ ability to create a smoke-free home. Fathers were keen to maintain their smoke-free home rules, and their motivations for this largely centered on their perceived role as protector of their children, and their desire to be a good role model.

**Conclusions:**

Fathers’ abilities to create a smoke-free home are shaped by a range of capabilities, opportunities, and motivations, some of which relate to their role as a father. Establishing a fuller understanding of the contextual and gender-specific factors that shape fathers’ views on smoking in the home will facilitate the development of interventions and initiatives that fathers can identify and engage with, for the broader benefit of families and to improve gender equity and health.

**Implications:**

Our findings can inform future development of father-centered and household-level smoke-free home interventions. They identify fathers’ views and experiences and help reframe smoking in the home as a gendered family-wide issue, which is important in building consensus on how best to support parents to create a smoke-free home. Our findings highlight the need for additional research to develop understanding of the ways in which gender-related aspects of family structures, heterosexual relationships, and child living arrangements influence home smoking rules and how to tailor interventions accordingly.

## Introduction

Supporting parents to create a smoke-free home is key to reducing children’s second-hand smoke (SHS) exposure. The smoke-free homes literature is dominated by interventions targeting mothers who smoke or who live in a home where an adult smokes.^1^ Research has identified the barriers and facilitators associated with mothers experiences of creating a smoke-free home, including women’s lack of agency in changing male smoking behaviors in the home.^2^ In contrast, father’s views and experiences are under-represented.^1^ Use of the term “father” in this paper is inclusive of men who are biological fathers, stepfathers, adoptive fathers, or who hold a significant caregiver role in the life of one or more children.

More than one-third (35%) of men in the world smoke, compared to just over 6% of women,^3^ and while there are no data on the proportion of fathers who smoke in the home global estimates suggest that nearly 50% of deaths from SHS occur among women and over 25% among children under five years.^4^ A recent Cochrane review^5^ of interventions to reduce children’s SHS exposure in the home found that only a third had a statistically significant effect. The authors were unable to identify what made interventions effective, suggesting no current consensus on how best to support parents to create a smoke-free home.

The call to include gender in tobacco control dates back nearly 40 years.^6,7^ Gender-sensitive approaches take into account the differences between men and women^8^ and have been used in Canada to develop father-friendly smoking cessation interventions.^9,10^ Gender-transformative approaches go further, using gender theory to design tobacco cessation/reduction initiatives with the dual aim of changing negative gender and social norms, and improving health and gender equity.^11,12^ Gender-transformative approaches are more often utilized in low and middle income countries to address the health issues of socially and economically marginalized groups and are currently less likely to be used to address mainstream health issues in higher income countries.^12^ Engaging with fathers in creating smoke-free homes for children is an emerging area of research, and an important step towards adopting gender-transformative approaches in tobacco control.^13^

Research on fathers’ involvement in creating smoke-free family environments has often focused on their role in supporting women to quit smoking during pregnancy, and the influence of their smoking status and attitudes on expectant mothers’ cessation.^14,15^ However, pregnancy provides an opportunity for both expectant mothers and fathers,^16^ and it has been suggested that shifts in masculinities as men become fathers should be considered in designing smoking cessation interventions for fathers.^17^ Children are also important agents in the smoking behavior change process, providing a strong motivation for fathers to quit smoking. Findings of a recent systematic review suggest the focus on fathers’ involvement in changing familial smoking behaviors should not be restricted to pregnancy and the post-partum phase.^16^

A recent scoping review^1^ identified only 12 studies published over the last 10 years that included findings on fathers’ roles in creating a smoke-free home, eight of which were conducted in Asian countries, three in Canada, and one in Turkey. Findings suggest that attitudes and knowledge, cultural and social norms, gendered power relations, and shifting perceptions/responsibilities related to fatherhood may influence fathers’ abilities to create a smoke-free home. However, male smoking rates and norms in Asian countries differ significantly from North America and Europe, where gender differences are less pronounced.^18^ Little is known about fathers’ experiences of creating/maintaining smoke-free homes in Western countries.^1^ Research is required to increase understanding of the barriers and facilitators that fathers face to inform the development of father-inclusive smoke-free home interventions. This would help reframe household smoking as a collective responsibility and a family-wide issue^19^ and address gender-specific issues underlying men’s smoking in the home. Engaging fathers in smoke-free homes initiatives could also lead to health gains for men, as creating a smoke-free home may reduce cigarette consumption and increase quit attempts.^20,21^

**Figure 1.**
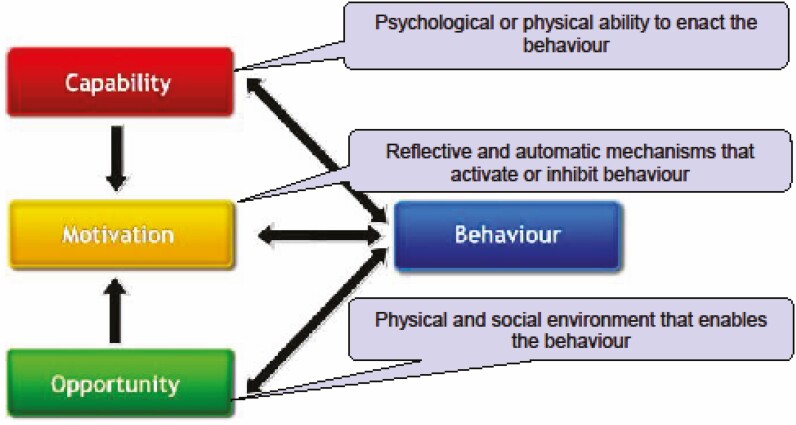
The COM-B model – a framework for understanding behavior change.^22^

The COM-B model^22^ (see Figure 1) proposes that for an individual to engage in a behavior they must have the capability, opportunity, and motivation to do so. Capability can be psychological or physical, opportunity can be social or physical, and motivation can be automatic or reflective. The COM-B model has been widely applied as a theoretical framework to explore diverse health behaviors including e-cigarette use^23^ and mothers’ home smoking behaviors.^19^ The aim of this research was to use the COM-B model to classify fathers’ barriers and facilitators to creating and maintaining a smoke-free home, to inform future father-inclusive smoke-free homes intervention development.

## Methods

### Recruitment

Fathers who smoked, lived with a partner (smoker or nonsmoker), extended family or on their own, and cared for one or more children aged 16 or under at least once a fortnight were eligible to participate. Fathers were asked “*Do you currently smoke cigarettes and/or rolling tobacco?*” at the time of initial contact. Additional information was then gathered on whether smoking sometimes took place in their home and if so, when, where, and by whom. Fathers who had a smoke-free home and those who smoked at home were invited to participate in interviews to explore barriers and facilitators. Participants were initially recruited through emails and subsequent prearranged visits to Early Years Centres (EYCs) (*n* = 5) and Dads community groups (*n* = 5) in Edinburgh to discuss the study and distribute participant information sheets. Emerging evidence supports the use of Facebook for recruitment,^24^ including from young/hard to reach groups^25,26^ and reaching fathers (especially young fathers) can be challenging.^27,28^ Based on our previous experience utilizing this approach,^29^ fathers (*n* = 8) were also recruited using paid Facebook advertisements to extend geographical/demographic reach. Fathers interested in participating after seeing the advertisement submitted their contact details securely online and were telephoned by one author (PM) to confirm eligibility. They were then emailed the participant information sheet, and after 48 h participation and interview arrangements were confirmed.

### Qualitative Interviews

Using a convenience sampling strategy, 20 fathers were invited to participate in interviews in their home, or in a quiet room within the EYC/community group venue, with one of the authors (PM). We considered this sample size feasible and sufficient to explore a range of fathers’ experiences. All were assured that the information they provided was confidential, and that they could withdraw at any stage during the interview and up to three months afterwards. Twenty fathers agreed to participate, two were uncontactable to arrange interviews. Written informed consent was obtained prior to each interview.

Interviews lasted approximately 80 min. Nuanced questioning techniques and reflexive interviewing strategies were used in line with existing guidance^30^ given dynamics of a male researcher conducting health-related interviews with fathers. The interview topic guide was based on smoke-free homes, men and tobacco use, and gender-transformative literature^1,2,11,31^ covering smoking history, current smoke-free home rules, changes to home smoking behaviors, including related to becoming/being a father, and perceived roles in creating a smoke-free home. Household negotiations involved in creating smoke-free home rules were also discussed and will be published separately. Participants received a £15 supermarket voucher. With participant permission, interviews were audio-recorded and transcribed verbatim. Ethical approval was granted by the University of Stirling’s General University Ethics Panel (GUEP 638).

### Qualitative Analysis

Reflexive thematic analysis was used given its focus on fluid coding processes and the importance of reflection on/engagement with the data.^32^ Anonymised transcripts were imported into NVivo 12 for coding. Two authors read each transcript, assigned inductive codes to text to describe potentially important features within the data, and noted possible interconnections. Transcripts were examined to identify the range and diversity of responses in relation to topics, and themes and subthemes were created and refined based on reexamining data and reflexive research team discussions. Themes were then mapped onto the core constructs of the COM-B model. Discrepancies regarding theme or interpretation were resolved by team members, including the most accurate descriptor where themes potentially mapped onto more than one COM-B construct.

The study was reported in accordance with the 32-item checklist of Consolidated Criteria for Reporting Qualitative Research (COREQ).^33^

## Results

Father’s names are presented as pseudonyms to protect anonymity. The characteristics of the 18 participating fathers are presented in Supplementary File 1. They were 32–46 years old. Eight lived with a partner; one separated father had sole physical custody and nine shared custody of their children with their ex-partner. Twelve fathers lived in areas in the bottom 40% Index of Multiple Deprivation (SIMD). Seven reported living in a smoke-free home, seven restricted home smoking to specific rooms, and four smoked in the home only when children weren’t present. All participating fathers were biological fathers to all the children they cared for in their home. The sections below explore fathers’ barriers and facilitators to creating/maintaining a smoke-free home in relation to the three COM-B model constructs. Themes are presented with illustrative quotes in Tables 1–3 alongside pseudonyms and home smoking status.

**Table 1.** Capability: Having the Physical and Psychological Skills to Create a Smoke-free Home

1. “If I fell out the back there would be nobody there to know that I’d fallen or hurt myself.” *(Martin, smoking allowed in bathroom)*
2. *“*Sometimes in the car I would’ve had a cigarette with [my child] in the back [pre-legislation] but… even though you think it’s [SHS] going out the window, it’s going everywhere and it’s quite detrimental to someone who doesn’t smoke…You can’t do it anymore, you’ve got to be responsible.” *(Andrew, smoking allowed if children not present)*
3. “I’ve got no idea…how much smoke could be escaping and travelling through to the living room, up the stairs, into her [child’s] bedroom. I couldn’t tell you about the science of it but I believe that we’re doing everything that we can….I believe we’re safe enough.” *(Liam, smoking is allowed in the dining room)*
4. *“*My ex-partner…it was a non-smoking house completely and I think that’s how none of my daughters smoke…So why am I not doing the same with my son? It is because of the stress…If I have been speaking to his mum sometimes and we have an argument, first thing I will do is come off the phone and have a fag…I just need to try and get back to the way I was…if I am not going to stop smoking then I need to take it out the house.” *(George, smoking allowed in kitchen)*
5. “When I’m feeling low I don’t want to go outside ‘cause I don’t want to bump into my neighbours… I can’t be bothered even getting up and going out so I’ve smoked in the kitchen.” *(Andrew, smoking allowed if children not present)*

**Table 2.** Opportunity: Environmental and Social Factors That Enable Steps Towards Creating a Smoke-free Home

1. *“*Hail, rain or snow I’ll stand outside…weather doesn’t really bother me. Plus I’ve got plans for building a wee shed up the back… like a kind of man cave where I can go out and have as many fags as I want...a ‘time out’ kind of place for me.” *(Fraser, smoke-free home)*
2. “People start smoking when they’re 9 years old here.” (*Paul, smoking allowed in bedroom)*
3. “My house my rules basically! That’s the way I see it. If they don’t like it then they don’t come to visit. I would just tell them straight. At the end of the day I’ve got to think about my daughter’s health and if they’re not prepared to think about that them they’re not really people that I want to have around.” *(Eric, smoking allowed if children not present)*
4. “Everybody just accepts it now. Even if it’s raining and some of the boys are up watching the football or whatever, it doesn’t matter. There are a few of us that smoke so we just go ‘ootside...Most of us have got kids to be fair.” *(James, smoke-free home)*
5. “You’d better no’ do it [smoke in front of children], because you know everyone will look and say ‘well that’s a bad parent’.’” *(Nathan, smoking allowed in kitchen)*
6. “I don’t like having a fag…if I’m on my own with the bairn in the buggy and I want a fag I won’t have one with her, but if I’m with my mate I’ll get my mate to push her and I’ll have one a distance away from the buggy. But I really hate that when I see people pushing a buggy and that and smoking a fag at the same time. (*Paul, smoking allowed in bedroom)*
7. “When I sit smoking, that’s my time, nobody really bothers me. I’ll just sit and do what want, that’s my free time basically…it’s kind of bad because then it puts me out the back instead of with my family all the time which is kind of a bad thing.” *(Fraser, smoke-free home)*

**Table 3.** Motivation: Automatic and Reflective Motivations That Affect Taking Steps Towards Creating a Smoke-free Home

1. “I do find myself watching the telly and I’ll have a wee fag…I just need to stop being so lazy and just go out there [outside to smoke].” *(George, smoking allowed in kitchen)*
2. “If I can’t get outside and I am climbing the walls with cravings…then I would nip into the bedroom and have a fag.” *(David, smoking allowed in bedroom)*
3. “You don’t let them close to the road when they’re young, you don’t let them into dangerous situations…so why should you smoke around your kids? I feel responsible for my daughter, and I know that smoking in the house is putting her in harm’s way, so I choose not to do it.” *(Andrew, smoking allowed if children not present)*
4. “As soon as you find out your wife or your partner is pregnant your mentality changes and it *did*…That was me having to go into the rain and the wind, or away into the kitchen [to smoke] whereas I used to light a fag up wherever I was sitting [in the home].” *(Robert, smoking allowed if no children present)*
5. “I want to make sure that my kids get what they want. You’re wasting £8 a day on cigarettes, £240 a month. That’s a holiday…It’s taking money that you could save for your kids for a rainy day. So I’m a selfish Dad.” *(Mark, smoke-free home)*
6. “So that…is probably the biggest source of guilt for me, that I’m doing something [smoking] that could shorten my life or limit my life in some way, and I’ve got a son there who might need looked out for, for the rest of his.” *(David, smoking allowed in his bedroom)*
7. “I see my role as a father as ‘do what I say don’t do what I do’. I try my best to not do things in front of my son that will damage his health.” *(George, smoking is allowed in kitchen)*

### Capability: Having the Physical and Psychological Skills to Create a Smoke-free Home

#### Physical Capability

Most fathers were physically able to take their smoking outside, with the exception of one with mobility issues, which affected his ability to navigate the steps from his house to outdoor garden space, influencing his decision to smoke in the home. He cared for his young child at home during the day whilst his partner was at work and was fearful of smoking outside (Table 1, Quote 1).

#### Knowledge of SHS-related Harms to Children

Fathers had limited contact with health visitors and GPs, who they recognized as a key source of SHS-related information and advice. Some suggested health professionals engaged more with mothers about SHS exposure at home given their status as the primary caregiver. However, fathers often displayed comprehensive knowledge regarding SHS risks, which they mostly attributed to media campaigns that had accompanied Scottish smoke-free legislation, in particular prohibiting smoking in cars carrying children (Table 1, Quote 2). A few still had limited SHS-related knowledge, including Liam whose decision to smoke in the dining room was guided by his belief that this was safe enough (Table 1, Quote 3).

#### Understanding of Effective and Ineffective Strategies

Several separated fathers modified their smoking rules according to part-custody arrangements, only smoking in the home when children were not staying with them. Others restricted smoking to one room that children used infrequently or smoked indoors only when children were a distance away (eg in bed upstairs). These fathers often took additional steps toward protecting their children from SHS (eg placing a rolled-up towel at the bottom of the door to minimize smoke-drift, having the oven extractor fan on). Whether these measures reduce children’s home SHS exposure is unclear, and whilst some fathers were confident their current strategies were effective, most fathers with partial smoke-free rules agreed they could be doing better (Table 1, Quote 4).

#### Mental Well-being

Personal mental health challenges sometimes presented a barrier to creating a smoke-free home, particularly for separated fathers. One father’s depression contributed to bouts of increased smoking indoors because he had reduced energy to go outside and interact with the outside world (Table 1, Quote 5). The stress associated with one father’s marital breakdown hindered his ability to create a smoke-free home, as he used smoking to alleviate stress (Table 1, Quote 4). Both acknowledged that smoking in the home was not effective in protecting their children.

### Opportunity: Environmental and Social Factors that Enable Steps Towards Creating a Smoke-free Home

#### Access to Outdoor Space and Weather Conditions

Most fathers had access to a private or shared garden space and did not view bad weather as a barrier to smoking outside. While some smoked in an outdoor shed or shelter others had aspirations to build a shed for smoking and to obtain “me-time” (Table 2, Quote 1).

#### Social Norms and Social Support

Fathers discussed smoking norms among family and friends and how these influenced their ability to maintain home smoking rules. A few fathers experienced pressure to relax rules by friends and family who smoked, and separated fathers sometimes experienced pressure from friends when their children were not with them. One father suggested this reflected how engrained smoking was where he lived (Table 2, Quote 2). Fathers occasionally referred to their home as their “castle”, adopting a “my house, my rules” approach (Table 2, Quote 3) where rules took precedence over the wishes of visiting friends and family who smoked. Some fathers said that visitors respected their home smoking rules, with one suggesting that support from fellow parents implied a shared understanding of the importance of maintaining a smoke-free home for children (Table 2, Quote 4).

#### Generational Shifts

Fathers generally viewed themselves as active, “hands-on” fathers, and often contrasted this to more traditional, distant roles their own fathers had played in their childhood. Fathers discussed generational shifts in smoking-related behaviors and attitudes, suggesting that whilst smoking near children was once acceptable, it was now stigmatized (Table 2, Quote 5). When fathers did take their children outside with them to enable outdoor smoking, the impacts of stigma were evident in their accounts (Table 2, Quote 6). Fathers who had created a smoke-free home spoke of feeling guilty sometimes when they smoked outside alone, considering this to be lost family time (Table 2, Quote 7).

### Motivation: Automatic and Reflective Motivations that Affect Taking Steps towards Creating a Smoke-free Home

#### Desires and Impulses

A few fathers noted that laziness, comfort, and/or boredom prevented them from creating a smoke-free home. George, who sometimes smoked in the living room in the evenings when his son was in bed, suggested this was a subconscious behavior rather than an active choice (Table 3, Quote 1). In contrast, David discussed his conscious decision to smoke in his bedroom when his child stayed with him, as he could not go outside and leave him alone indoors. This only happened if his cravings felt insurmountable (Table 3, Quote 2).

#### The Father-protector Role

Fathers held strong beliefs about their responsibility to protect their children from harm. This “father-protector” role, often interlinked with the notion of being a “good” father, was a key motivator for reducing children’s home SHS exposure (Table 3, Quote 3). In a few cases, fathers attributed changes in home smoking rules to becoming a father, signaling their shift to the father-protector role and a new focus on their responsibilities as a father (Table 3, Quote 4).

#### Fathers as a Positive Role Model

Fathers were motivated to be positive role models for their children, and consequently often wrestled with their nicotine addiction, unsuccessful attempts to stop smoking (viewed as embarrassing or a failure), and the financial impact of smoking on the family, which one father described as selfish (Table 3, Quote 5). Fathers were conscious of the long-term health risks of smoking, which clashed with their visions of fathering children into adolescence and adulthood, causing them guilt (Table 3, Quote 6). Creating a smoke-free home and keeping smoking out of children’s sight sometimes helped to resolve guilt and alleviate fears that children might become smokers too. However, a few fathers took a *“do what I say, not as I do*” approach, which facilitated a more relaxed approach to smoking in the home, helping to legitimise their home smoking rules (Table 3, Quote 7).

## Discussion

Fathers’ abilities to create and maintain a smoke-free home were shaped by a range of capabilities (knowledge and awareness of effective strategies); opportunities (generational shifts, nonsmoking social norms, supportive friends and family), and motivations (becoming a father, the father-protector role, a positive role model). These findings add to the limited research on fathers’ experiences of creating a smoke-free home.^1^ They support Canadian findings on how shifting perceptions and responsibilities related to fatherhood^34,35^ influence fathers’ home smoking behaviors. Positive role modeling was a more prominent motivator for protecting children from second-hand smoke in our study, alongside the role of the father as protector. This may reflect the life stage of several participants who had older children and were not “young” fathers. Further research is required to compare nuanced differences in motivations associated with parental home smoking behavior change at different stages of familial development.

Our findings support the suggestion that beliefs and knowledge about SHS can shape fathers’ home smoking behavior,^1,36,37^ which Passey et al.^2^ suggest is the case for mothers. Fathers viewed public health campaigns as their primary source of knowledge in the absence of established relationships with health professionals regarding their children’s health. This highlights the importance of public health messaging as a means of reaching and engaging fathers, given they tend to be considered hard to reach.^27^

Our findings differ from those of Passey et al.^2^ who report that *“many participants* [women]…*were unwilling to or uncomfortable asking visitors not to smoke inside”* (pp. 10–11), reflecting cultural practices, gendered power imbalances, or respect for visitors’ need to smoke. By contrast, the fathers in our study generally presented themselves as confident to enforce home smoking rules reflecting traditional masculine ideals of the man being the “head of the household” and enforcer of household rules – even when they had moved out of the family home. Some went further to ensure their home stayed smoke-free, building outdoor shelters/using sheds, choosing to smoke in public with their children despite feeling stigmatized, and opting to smoke outdoors away from their children, despite feeling guilty about lost family time. They were generally committed to minimizing their children’s exposure to SHS in the home, motivated by their father-protector/role model beliefs. These findings can inform the development of gender-sensitive, father-centered smoke-free home interventions, using an approach similar to the Canadian “Dads in Gear” programme, which provides peer support to fathers using evidence-based intervention components, goal setting, and discussion activities as they reduce and stop smoking.^38^ Specific approaches and content could be codesigned with fathers to ensure that positive masculine ideals utilized within gender-sensitive programmes are appropriate to the culture and context of the target population.^39^

Fathers who had partial smoke-free home rules often noted that they “*could be doing better”.* This highlights the importance of utlizing nonjudgmental, empathic approaches in smoke-free homes interventions, using asset based approaches that harness, build on, and value steps already made.^2,40^ In cases where additional change is feasible to create a smoke-free home, interventions that encourage participants to plan coping responses could also be developed, such as listing potential barriers and ways to overcome them, identifying high-risk situations, and practicing coping responses. This approach is supported by research that also suggests that professionals should be trained to develop skills in advising on practical smoke-free home strategies.^2,41^

As our findings indicate that fathers are active agents in creating smoke-free homes, household-level interventions that engage fathers and mothers may prove effective in facilitating shifts to smoke-free homes. However, such interventions should be cognizant of unequal familial power relations between men and women and make attempts to ameliorate them. Systems-based approaches such as SystemCHANGE that focus on the redesign of family daily routines to support healthy lifestyles,^42^ blended with gender-transformative content and messaging that reframes masculine interpretations of fathering to include protection, nurturing, and caring, could be explored. This would assist both fathers and mothers, living together or apart, to create and maintain a smoke-free home. Previous research suggests that tobacco can play a critical role in couple interactions, routines, and relationships, and that partner smoking status influences women’s smoking reduction and vice versa.^43^ Interventions targeting spouses or cohabiting partners could include a focus on these interactions to better understand smoking practices in the home and increase partner support for smoking behavior change. This is especially important given that existing interventions aimed at enhancing partner support for smoking cessation are ineffective.^44^

Developing future smoke-free homes interventions using a gender-transformative approach would seek to benefit the health of both men and women simultaneously, and shift the social values and cultural norms associated with smoking in the home. This approach could be particularly relevant for the East Asia and Pacific region where 49% of men smoke compared to under 3% of women.^3^ It could incorporate education that encourages reflection on the impact of prevailing gendered norms on home smoking behaviors and associated health outcomes, highlighting how gendered norms can change. This approach has been used in previous gender-transformative health promotion programmes, alongside the involvement of men and women in the planning and delivery of programme interventions.^12^

In this study, separated fathers often discussed the fluidity of their home smoking rules in relation to part-time custody arrangements, only smoking in their home on days when their children were absent. They more often spoke about challenges associated with depression, stress, and lone parenting which presented barriers to creating a smoke-free home. In addition, some received pressure from friends to relax smoke-free home rules when their children were absent. These findings are interesting given data from the United States and Canada^45,46^ suggesting that single parents, regardless of income or neighborhood deprivation are less likely to report smoke-free home rules. In the United Kingdom, approximately 25% of families with dependent children are single-parent families (including families where part-custody arrangements are in place).^47^ Restricting home smoking to days when children are not living in the home may present a practical solution for some fathers in reducing their children’s SHS exposure,^48^ but exposure to nicotine-laden dust and vapor reemissions from furnishings (“third-hand smoke”) could still be an issue when children visit,^49^ making this approach short of the ideal of a completely smoke-free home. Additional research is required to build a full understanding of the ways in which family structures, the breakdown of heterosexual relationships, and child living and custodial arrangements influence home smoking rules. Our findings support previous suggestions that single parents may represent a distinct subgroup that requires targeted intervention to enable smoke-free homes.^45^

We are not aware of other UK/European smoke-free home studies that have specifically focused on fathers. Our findings present new insights that can form the basis for developing interventions to actively involve and appeal to fathers and create gender-specific and transformative health promotion messages on home smoking. Half the participants in this study were separated fathers whose children did not live in the same home as them each day. Their perceptions regarding the importance of creating a smoke-free home sometimes differed from fathers who lived with their children full-time. However, their inclusion in this study ensured that we captured a broader range of fathers’ views and experiences. Our sample comprised White fathers, and did not include younger fathers, despite efforts to broaden recruitment. Whilst the COM-B model facilitated the classification of barriers and facilitators, categorizing themes that potentially mapped onto more than one COM-B construct proved challenging, as reported previously.^23^ As our findings represent the views of a relatively small number of (biological) fathers, and we acknowledge that other fathers may experience additional barriers and facilitators not raised here.

Fathers have an important role to play in creating a smoke-free home, and our findings provide insights regarding their capabilities, opportunities, and motivations for doing so. Establishing a fuller understanding of the contextual and gender-specific factors that shape fathers’ experiences of creating and maintaining a smoke-free home will facilitate the development of father-inclusive interventions and initiatives that address and transform masculine conceptions of fathering, acknowledge unequal power relations between men and women in relationships and aim for the broader benefit of improving gender equity and health.

## Supplementary Material

A Contributorship Form detailing each author’s specific involvement with this content, as well as any supplementary data, are available online at https://academic.oup.com/ntr.

ntab228_suppl_Supplementary_Materials

ntab228_suppl_Supplementary_Taxonomy_Form

## Data Availability

The anonymised data underlying this article may be shared on reasonable request to the corresponding author following review.
