# Supplementary material for: What Helps and What Hinders the Creation of a Smoke-free Home: A Qualitative Study of Fathers in Scotland
Source: Nicotine Tob Res. 2021 Nov 10;24(4):511–8. doi: 10.1093/ntr/ntab228 (PMC8887574; doi:10.1093/ntr/ntab228)
Supplement: ntab228_suppl_Supplementary_Materials [file ntab228_suppl_supplementary_materials.pdf]

Supplementary file 1: Characteristics of study participants

| Participant Pseudonym | SIMD* | Age | Marital status** | Employment Status | Home Type | Garden | Smoking Per Day | Children | Home Smoking Rule | Other Home Smokers |
|---|---|---|---|---|---|---|---|---|---|---|
| Andrew | 3 | 37 | Separated | Unemployed | Flat 1st Floor | Shared Garden | up to 10 | 1 (age 15) | Smoking is allowed if no children present | 0 |
| Brian | 1 | 45 | Single | Employed FT | Flat 1st Floor | Shared Garden | More than 20 | 3 (ages 2, 3 and 15) | Smoking is not allowed | 0 |
| Charlie | 1 | 34 | Single | Other (Volunteer) | Flat Ground Floor | Shared Garden | Between 10-20 | 1 (age 4) | Smoking is not allowed | 0 |
| David | 1 | 43 | Divorced | Employed PT | Flat 1st Floor | No Garden | Between 10-20 | 1 (age 8) | Smoking is allowed in his bedroom | 0 |
| Eric | 1 | 32 | Single | Employed PT | Flat 1st Floor | Private Garden | Between 10-20 | 1 (age 9) | Smoking is allowed if no children present | 0 |
| Fraser | 1 | 32 | Living with partner | Employed FT | Terraced House | Private Garden | More than 20 | 1 (age 3) Partner pregnant | Smoking is not allowed | 0 |
| George | 4 | 47 | Single | Unemployed | Terraced House | Private Garden | Between 10-20 | 1 (age 10) | Smoking is allowed in the kitchen | 0 |
| Harry | 2 | 44 | Separated | Employed FT | Flat 1st Floor | Shared Garden | Between 10-20 | 4 (ages 3, 5, 9 and 15) | Smoking is allowed if no children present | 0 |
| Ian | 2 | 35 | Single | Unemployed | Flat 3rd Floor | Shared garden | Between 10-20 | 2 (ages 9 and 13) | Smoking is allowed if no children present | 0 |
| James | 1 | 33 | Married | Unemployed | Flat 1st floor | Shared garden | Between 10-20 | 1 (age 2) | Smoking is not allowed | 0 |

| | | | | | | | | | | |
|---|---|---|---|---|---|---|---|---|---|---|
| Kenny | 3 | 45 | Separated | Prefer not to say | Flat 2nd floor | Private garden | Between 10-20 | 1 (age 6) | Smoking is not allowed | 0 |
| Liam | 5 | 34 | Married | Employed FT | Semi-detached | Private Garden | up to 10 | 1 (age 7) | Smoking is allowed in the dining room | 1 (Wife, less than 10 a day) |
| Martin | 1 | 35 | Married | Employed FT | Flat ground floor | Shared garden | Between 10-20 | 2 (ages 3 and 16) Partner pregnant | Smoking is allowed in the bathroom | 0 |
| Nathan | 2 | 34 | Married | Unemployed | Semi-detached | Private garden | Between 10-20 | 2 (age 4 and 2) | Smoking is allowed in the kitchen (and also the living room when no children present) | 1 (Wife, number of cigarettes smoked per day not raised during interview) |
| Mark | 2 | 46 | Living with partner | Employed PT | Semi-detached | Private garden | Between 10-20 | 3 (ages 2, 14 and 18) | Smoking is not allowed | 1 (Partner, between 10-20 a day) |
| Peter | 3 | 46 | Living with partner | Unemployed | Semi-detached | Private garden | Up to 10 | 1 (age 2) | Smoking is not allowed | 0 |
| Paul | 2 | 37 | Single | Unemployed | Flat 3rd floor | Shared garden | Up to 10 | 1 (age 2) | Smoking is allowed in his bedroom | 0 |
| Robert | 3 | 34 | Married | Self-employed | Terraced house | Private garden | Up to 10 | 1 (age 2) | Smoking is allowed if no children present | 0 |

*estimated using the Scottish Index of Multiple Deprivation (SIMD) look up tables. SIMD quintiles range from 1 – 5, with 1 indicating the most deprived areas in Scotland, and 5 indicating the most affluent areas in Scotland.

**all fathers who were single, separated or divorced lived with no other adult in their home.
